# An automated quantitative analysis of cell, nucleus and focal adhesion morphology

**DOI:** 10.1371/journal.pone.0195201

**Published:** 2018-03-30

**Authors:** Antonetta B. C. Buskermolen, Nicholas A. Kurniawan, Carlijn V. C. Bouten

**Affiliations:** 1 Department of Biomedical Engineering, Eindhoven University of Technology, Eindhoven, The Netherlands; 2 Institute for Complex Molecular Systems, Eindhoven University of Technology, Eindhoven, The Netherlands; Pennsylvania State Hershey College of Medicine, UNITED STATES

## Abstract

Adherent cells sense the physical properties of their environment via focal adhesions. Improved understanding of how cells sense and response to their physical surroundings is aided by quantitative evaluation of focal adhesion size, number, orientation, and distribution in conjunction with the morphology of single cells and the corresponding nuclei. We developed a fast, user-friendly and automated image analysis algorithm capable of capturing and characterizing these individual components with a high level of accuracy. We demonstrate the robustness and applicability of the algorithm by quantifying morphological changes in response to a variety of environmental changes as well as manipulations of cellular components of mechanotransductions. Finally, as a proof-of-concept we use our algorithm to quantify the effect of Rho-associated kinase inhibitor Y-27632 on focal adhesion maturation. We show that a decrease in cell contractility leads to a decrease in focal adhesion size and aspect ratio.

## Introduction

In the last decades studies have shown the essential role of cell adhesion in processes like cell migration [1], survival, proliferation, and differentiation [2], as well as tissue morphogenesis [3]. These types of cell behavior are affected by the physical properties from the cell micro-environment as adherent cells have the ability to sense and respond to these properties by adapting their shape and orientation. More specifically, signals from the micro-environment are transmitted to the interior of the cell through a structural pathway, i.e. focal adhesions (FAs) physically linking the environment via the actin cytoskeleton to the nucleus. Although intense efforts have been devoted to understand how cells sense and respond to the properties of the micro-environment via FAs, the functional underlying mechanisms are not yet fully understood [4].

FAs consist of a large number of proteins, such as vinculin, paxillin, focal adhesion kinase (FAK) and talin, and can range from 0.2 μm to 30 μm in size depending on the maturation stage of the FA as well as the cell type [5]. Within the same cell, diverse types of adhesion structures can be present, including small, round nascent focal adhesion structures (e.g., focal complexes), larger focal adhesions, and more stable fibrillar adhesions. These adhesion types differ morphologically, molecularly, and dynamically [6]. The maturation of nascent FAs into larger, elongated, FAs is dependent on the bundling of actin filaments and the generation of mechanical force by myosin II activity [7, 8]. A principal mediator of myosin II activity is the small GTPase RhoA and its downstream effector Rho-associated kinase (ROCK). The activation of myosin II leads to the accumulation of activated myosin motor proteins, which bind the actin filaments to create adhesion-associated actin bundles called stress fibers. Accordingly, ROCK activation promotes actin-myosin mediated contractile force generation, formation of stress fibers, and morphological changes in FAs [9–12]. To examine the role of certain cell properties on specific (sub)cellular morphological features, investigators commonly treated the cells with pharmacological drugs that can interfere with certain protein properties. This has resulted in a wealth of information on how cells respond to physical aspects of the micro-environment to improve the mechanistic understanding on cellular mechanosensing. Yet, comparison of the results of different investigations can be challenging because of the complexity of the cellular response, which depends on the cell type and the choice of physical experimental parameters [13]. An unbiased, quantitative analysis of cellular, nuclear and FA morphological changes can aid in the further uncovering of the mechanisms behind cellular responses to physical properties of the environment.

Recent advances in imaging techniques available to cell biologists, including new labeling methods and microscope designs, have made it possible to visualize various aspects of cellular behavior in more detail [14]. However, visual inspection of cellular immunofluorescence images is often insufficient to detect or describe subtle but important changes due to lack of objectivity and reproducibility. To adequately characterize these subtle changes, an automated, quantitative assessment is often desired. A number of automated image analysis algorithms have been developed for analysis of immunofluorescence images for (semi-) automated cell segmentation [15–18] or detection and characterization of FAs [19–23]. S1 Table shows an overview of these studies. Despite these approaches, there is a lack of access to a user-friendly, automated, unbiased image analysis tool that is able to detect changes in the combination of cellular, nuclear, and FA morphological features in a robust and accurate way.

**Fig 1:**
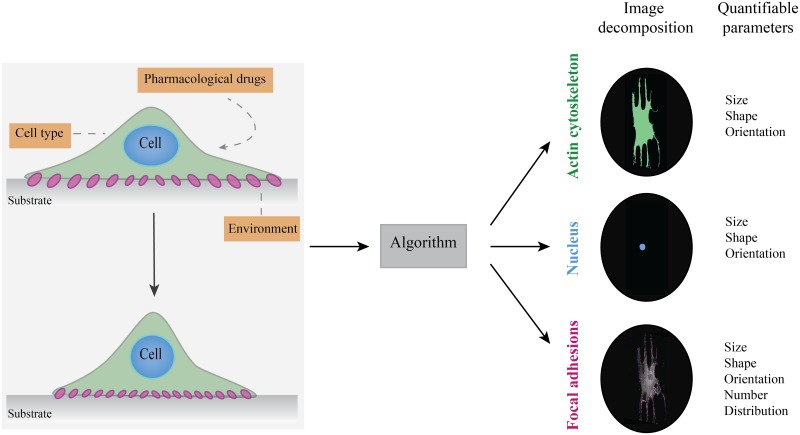
Schematic overview of the morphometric features of the cell, nucleus, and focal adhesions (FAs), providing information about the effects of cell type, physical environment, and pharmacological drugs on cell response. The cell type, physical properties of the environment, and pharmacological drugs are known to affect cellular, nuclear and FA morphology. With the developed algorithm we were able to detect these changes and translate them into quantifiable parameters.

This paper describes the development and evaluation of a novel automated algorithm for the segmentation and quantification of single cells, nuclei and individual FAs. To demonstrate the validity of the designed algorithm, we tested the platform on myofibroblasts seeded on fibronectin-coated substrates to automatically detect individual cells, nuclei, and FAs from immunofluorescence images. We illustrated the broad applicability of our algorithm (Fig 1) by showing that we were able to detect differences in morphological features between different cell types, environments, and in response to pharmacological drugs. Finally, using our algorithm we demonstrated that we were able to quantify the influence of ROCK inhibitor Y-27632 on FA morphology, demonstrating the direct effect of Rho-mediated cell contractility on FA maturation in myofibroblasts. Taken together, the developed algorithm enables successful segmentation and quantification of single cells, nuclei and individual FAs and can be used to obtain interpretable quantitative data essential to identify important morphological changes in response to diverse factors affecting (sub)cellular morphology.

## Materials and methods

Algorithms to quantify cellular, nuclear and focal adhesion morphology were developed and optimized using a primary cell source, Human Vena Saphena Cells (HVSCs). To test the robustness and applicability of the method, the algorithms were subsequently applied and evaluated using a second cell type, under pharmacological manipulation, and substrate manipulation. All cell culture and manipulations were performed at 37°C in 5% CO_2_.

### Experimental design

#### Cell culture

HVSCs were harvested from the human vena saphena magna according to the Dutch guidelines for secondary use of material (kindly provided by the Catharina Hospital Eindhoven) and have previously been characterized as myofibroblasts [24]. The HVSCs were cultured in advanced Dulbecco’s Modified Eagles Medium (DMEM) (Invitrogen, Breda, The Netherlands) supplemented with 10% Fetal Bovine Serum (Greiner Bio-One, Alphen aan den Rijn, the Netherlands), 1% penicillin/streptomycin (Lonza, Basel, Switzerland), 1% GlutaMax (Invitrogen, Breda, The Netherlands). Only HVSCs with a passage lower than 7 were used in this study. HVSCs were seeded at a density of 2000 cells/cm^2^. Mouse Embryonic Fibroblasts (MEFs, kindly provided by Prof. Cecilia Sahlgren, Åbo Akademi, Turku, Finland) were cultured in Advanced Dulbecco’s Modified Eagle’s Medium (DMEM; Gibco 12491) supplemented with 5% fetal bovine serum (Greiner Bio-one), 1% L-glutamine, and 1% penicillin/streptomycin (Lonza) (Life Technologies). MEFs were passaged 1:3 every two days and a minimum of three times before seeding. MEFs were seeded at a density of 10000 cells/cm^2^. Both cell types were cultured for 24 hours on glass substrates homogeneously coated with fibronectin. For this, the glass coverslips were sterilized with 70% ethanol for 5 minutes, whereafter the coverslips were incubated with 50 μg/ml of fibronectin from human plasma (Sigma-Aldrich, St. Louis, USA) for 1 hour at room temperature. The substrates were rinsed at least three times with phosphate buffered saline (PBS) and immersed in PBS until cells were ready for seeding.

#### Pharmacological manipulation

A pharmacological inhibitor of the Rho-associated kinase (ROCK) pathway, Y-27632 (Sigma-Aldrich), was diluted in stock dimethyl sulfoxide (DMSO) and was then added to the culture medium for final drug concentrations of 20 μM, 10 μM, and 5 μM. Cells were plated for 23 hours and then incubated for 1 hour in Y-27632 before fixation. Control experiments were performed with drug-free medium containing DMSO at the same concentration as in the drug medium to assure that DMSO was not affecting the cells.

#### Micropatterned substrates

The micropatterned substrates consisting of 5 μm lines with 5 μm inter-line spacing were fabricated via standard photolithography techniques, according to previous protocols [25]. Briefly, the polydimethylsiloxane (PDMS) stamp was incubated with 50 μg/ml of fibronectin (Cytoskeleton, Denver, CO, USA) for 1 hour at room temperature. The stamp was then dried and put in contact with a PDMS coated coverslip for 15 minutes to imprint the pattern. Uncoated regions were blocked by immersing the micropatterned coverslips for 5 min in a 1% solution of Pluronic F-127 (Sigma-Aldrich, St. Louis, USA). Finally, the coverslips were three times washed with PBS and stored in PBS at 4°C until use.

#### Visualization by immunofluorescence analysis

For the visualization of the actin cytoskeleton, the nucleus, and focal adhesions (FAs) the cells were fixed for 15 minutes with 4% formaldehyde (Sigma-Aldrich, St. Louis, USA) in PBS 24 hours after cell seeding. After this, the cells were permeabilized with 0.5% Triton-X-100 (Merck, Amsterdam, The Netherlands) for 15 minutes and blocked for 30 minutes with 4% goat serum in PBS at room temperature. Coverslips were then incubated with the primary anti-vinculin antibody IgG1 (V9131, Sigma-Aldrich, St. Louis, USA) overnight at 4°C for staining the FAs. Subsequently, samples were rinsed three times in 0.05% Tween-PBS and incubated for 1 hour with Alexa Fluor 647 goat anti-mouse (Molecular Probes) 1:500 and FITC-conjugated phalloidin (15500, Phalloidin-Atto 488, Sigma-Aldrich, St. Louis, USA) 1:200 for staining the actin cytoskeleton. Finally, the samples were incubated with DAPI (Sigma-Aldrich, St. Louis, USA) for 5 minutes for immunofluorescence of the nucleus and mounted onto glass slides using Mowiol (Sigma-Aldrich, St. Louis, USA). The images of the cells were acquired using an inverted microscope (Zeiss Axiovert 200M, Zeiss, Gottingen, Germany), using 20 x/0.25, 40 x/0.25 objectives (HVSCs) or 100x/1.25 objective (MEFs). All images were exported as TIFF files.

### Image analysis algorithm

**Fig 2:**
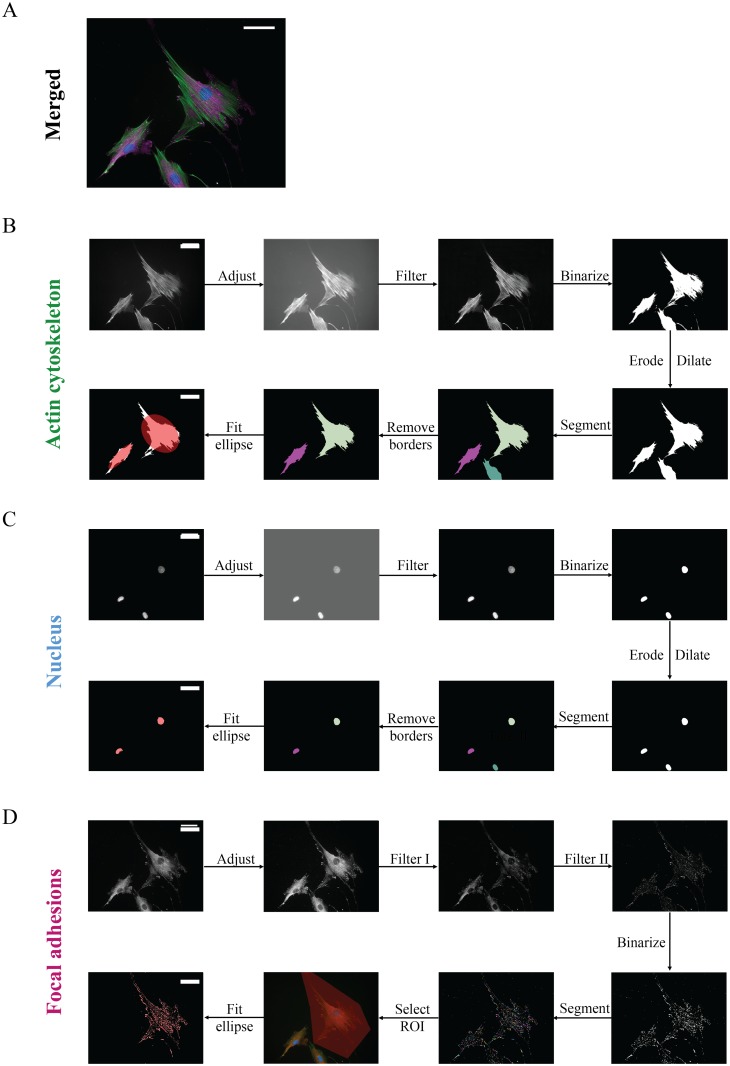
Overview of the steps of the automated image analysis pipeline. Representative immunofluorescent image of Human Vena Saphena Cells (HVSCs), (A) stained for the actin cytoskeleton (green), nucleus (blue), and focal adhesions (magenta). To automatically detect and analyze cells (B), nuclei (C) and focal adhesions (D), corresponding grey-scale images were processed using the automated image analysis pipeline. Scale bars: 50 μm.

We developed a pipeline for automated image analysis designed to detect single objects (e.g. cells, nuclei, and FAs) and extract their morphological features (Fig 2). For this, the acquired grey-scale images of the actin cytoskeleton, nucleus and FAs were processed and analyzed using a custom-made code written in Mathematica 11.1 (Wolfram Research, Inc., Champaign, USA), which is available as a supplementary file S1 File.

#### Cell and nucleus detection

To detect and analyze single cells and their corresponding nuclei, first the contrast of the grey-scale images of the actin cytoskeleton and the nuclei were adjusted by redistributing the pixel values to cover a range between 0 to 1. To enhance the objects of interests (e.g. cells and nuclei) and suppress the effect of any non uniform illumination, a top-hat filtering was applied to the images. Then, the images were binarized automatically by using Otsu’s method [26]. To fill all holes (regions of background completely enclosed by object) in the binarized images, the detected objects were filled, followed by a closing operation. This operation includes a dilation (1x1 box mask, 2r+1 x 2r+1, with r = 1) followed by an erosion (1x1 box mask) operation on the binary representation of the cells or nuclei. After this, individual cells and their corresponding nuclei were segmented by subjecting all the processed images to a marker-controlled watershed segmentation algorithm. For this, markers were generated by using a distance transform. The distance transform converts the binary image, consisting of foreground and background pixels, into a distance map where the distance from every pixel of the object component (foreground pixel) to the nearest background pixel was determined. The regional maxima of the inverse of the distance map were regarded as markers, where all maxima with values smaller than a threshold value *h* were suppressed. The exact value of *h* depends on the image and experimental settings (e.g. objective, cell type) and needs therefore to be adjusted for every new set of settings to minimize the number of misdetections. Cells touching the image boundaries and small objects that fail the size criterion to be a single cell (objects smaller than 20 μm^2^) were automatically excluded from analysis. The remaining, segmented images of the cells were multiplied with the segmented images of the nuclei, to be left with only the corresponding nuclei to the cells. Fig 2B and 2C show the examples of the processed and segmented images for the cells and nuclei, respectively.

#### Focal adhesion detection

To test the automated segmentation of the focal adhesions (FAs), here we analyzed grey-scale images of vinculin, which we chose as a marker for FAs, however, other markers of FAs such as paxillin or focal adhesion kinase could also be used. The presence of soluble vinculin in the cytosol, which can lead to a strong background signal, can be intensity-discriminated using previously-proposed methods [23, 27]. Before quantification of the FAs, the image contrast of the grey-scale images was adjusted by redistributing the pixel values to cover a range between 0 to 1 (Fig 2D, ‘Adjust’). Then the images were subjected to a two-step filtering process to both suppress the effect of the soluble vinculin in the cytosol and enhance the signal from individual FAs, using a top-hat filtering and median filter, respectively. The top-hat filtering applies a structuring element (a small disk with a diameter of 80 pixels) on the input image to find objects (e.g. FAs) that are smaller than the structuring element and brighter than their surrounding background. Subsequently, we applied a median filter with a size of 4 μm x 4 μm for both HVSCs and MEFs to determine the residual background of the image. This background image was subtracted from the original image. The size of the kernel of the median filter was determined by taking the pixel width of the largest FAs based on the original image, which may depend on the cell type. The remaining images (Fig 2D, ‘Filter II’) were further automatically processed using Otsu’s method [26] to obtain binarized images. The robustness of the detection of FAs with this thresholding method was confirmed qualitatively by visual inspection across a large number of images of varying image quality (e.g. different levels of background). After this, individual FAs were segmented by replacing each pixel by an integer index representing the connected foreground image component in which the pixel lies. The segmented FAs were marked by a random color to identify each single FA (Fig 2D, ‘Segment’). From this the FAs were extracted automatically to analyze FAs of multiple cells. To analyze the FAs of only one single cell, the cell of interest was selected by tracing the cell using a polygonal mask to select the region of interest (ROI)(Fig 2D, ‘Select ROI’). This polygonal mask could be adjusted manually as necessary. FAs smaller than 0.1 μm^2^ were excluded to ensure that background noise is eliminated from the analysis.

#### Morphological features

Based on the segmentation of each identified object (e.g. cell, nucleus and focal adhesion), a large number of features could be determined, including area, orientation, aspect ratio (corresponding short and long axis), length, and perimeter. To determine the orientation and aspect ratio of the object, binarized images of the profile of each object were automatically fitted with an ellipse. The orientation was defined as the angle of the major axis of the ellipse and the aspect ratio was defined as the ratio of the ellipses major axis to its minor axis. Furthermore, we analyzed the spatial distribution of the FAs by calculating the Euclidean distance between the centroid of the nucleus and the centroids of FAs. All parameters were calculated from individual segmented cells, nuclei, or FAs using the Mathematica ComponentMeasurement function with the properties specified as: area, orientation, length, width, Feret diameter and perimeter, respectively.

## Results and discussion

To demonstrate that the algorithm could automatically and accurately detect morphological changes in cells, nuclei and focal adhesions (FAs) rapidly, we first cultured Human Vena Saphena Cells (HVSCs) on homogeneously coated substrates with fibronectin and determined the morphological features of the cell, nucleus and focal adhesions (FAs). Accordingly, the robustness and applicability of the algorithm was tested by comparing three well-known factors affecting cellular, nuclear, and focal adhesion morphology to see whether the algorithm was able to detect changes in morphological features. Then as a proof-of-concept, the new algorithm was applied to quantify the morphological features of the FAs in response to a pharmacological drug which is known to inhibit a modulator of contractility.

### Validation of automated detection of cells, nuclei and focal adhesions

**Fig 3:**
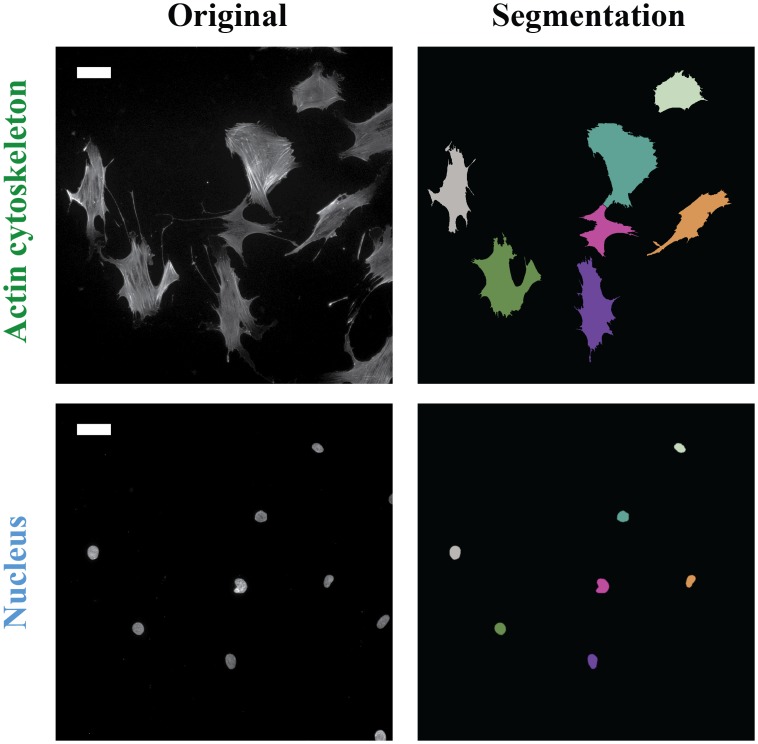
Automatic cell and nucleus segmentation. Representative immunofluorescence images of the actin cytosketeleton and nuclei and the corresponding segmentation results, with each identified cell and nucleus shown in a different color. Scale bar: 50 μm.

We first investigated the performance of the algorithm to detect 1) single cells and nuclei and 2) individual FAs. To locate individual cells and nuclei, the processed images were subjected to a segmentation algorithm. As shown in the review of Wiesmann et al. [28], where they performed an image processing comparative study with 15 freely available segmentation tools on four representative fluorescence images, cell separation was not possible with most of the available tools. In these tools, watershed-based algorithms were used to segment individual objects, however, a well-known drawback is over-segmentation [17]. It has been shown that marker-controlled watershed segmentation can effectively solve over-segmentation problems [29]. Therefore, we proposed a marker-controlled watershed segmentation-based algorithm in which the markers were automatically determined and where we could automatically detect individual objects. Fig 3 shows that our algorithm was able to successfully segment individual cells and their corresponding nuclei: cells and nuclei were correctly separated and the objects touching the edge of the image were removed.

**Fig 4:**
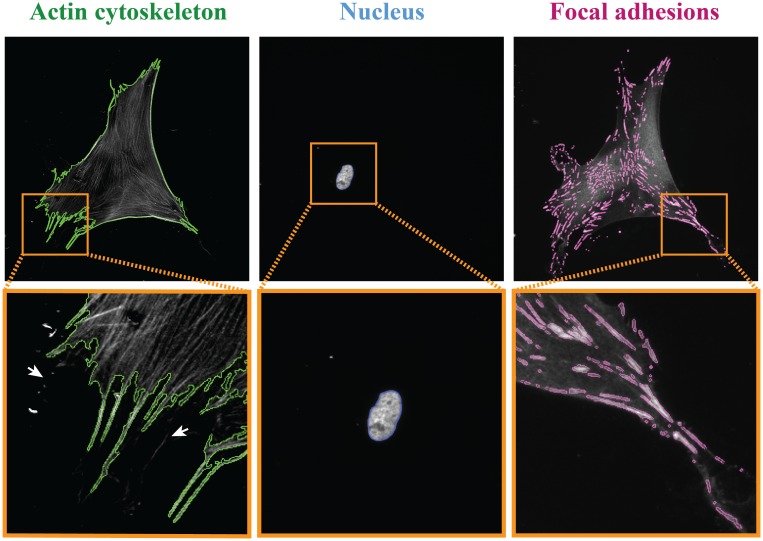
Detection of a single cell, nucleus and focal adhesions (FAs). Representative grey-scale images of the actin cytoskeleton, nucleus, and FAs of HVSCs on a substrate homogeneously coated with fibronectin. The detected outlines are shown in green, blue, and magenta, respectively, and the orange rectangles marked areas show zoom-in images of the cell, nucleus and FAs. The white arrows indicate some small actin-rich membrane protrusions that were not detected.

To confirm accurate detection of single cells and their corresponding nuclei, Fig 4 shows a representative example of a Human Vena Saphena Cell (HVSC) stained for the actin cytoskeleton, nucleus and FAs with the overlays of their detected outlines in green, blue, and magenta respectively. The detected outlines acquired from the actin cytoskeleton and nucleus images are in good agreement with the cell and nucleus boundary, demonstrating that the algorithm was able to discern cells and nuclei accurately. The zoomed-in image of the cell showed that smaller, localized actin-rich membrane protrusions could also be detected. Although localized membrane protrusions with a low fluorescent intensity could not always be detected by our algorithm (white arrows), we expect that these features will be too small to influence the overall morphological features of the cell. An automated analysis of these protrusions itself was not included in the present study, but can be determined with the algorithm presented by Barry et al. [30].

Next, we used algorithm to detect individual FAs. Often, raw grey-scale vinculin images are noisy, displaying diffuse staining in the cytoplasm as for example shown in Fig 2D. We designed a two-step image-filtering procedure to remove the noise originating from the soluble vinculin in the cytoplasm and to segment the FAs. The detected outlines of the FAs shown in Fig 4 demonstrate that our proposed algorithm was capable of distinguishing FAs that are close to or even touching each other and that the segmentation method successfully identified individual adhesions. To minimize false positives from unspecific staining of other structures with a high fluorescent intensity, we defined a threshold (30 μm) for the length of individual FAs based on the length of all analyzed FAs (S1 Fig). Structures larger than this threshold were not included in the quantitative analysis of the FAs.

We note that our proposed image analysis pipeline allows fast analysis of morphometric features of cells, nuclei and FAs. As an indication, analyzing a dataset of 20 images per staining (e.g. fibronectin, actin cytoskeleton, nucleus, and vinculin) can be completed in about 10 minutes using a standard PC with a Core i5-4670 processor and 8 GB RAM. This, together with the user-input-free feature detection, enables a high-throughput quantitative analysis of a series of images in an unbiased manner, which is useful for analyzing a large number of images (e.g. time-lapse) and for objective comparison across different experimental conditions.

### Robustness and applicability of the algorithm

**Fig 5:**
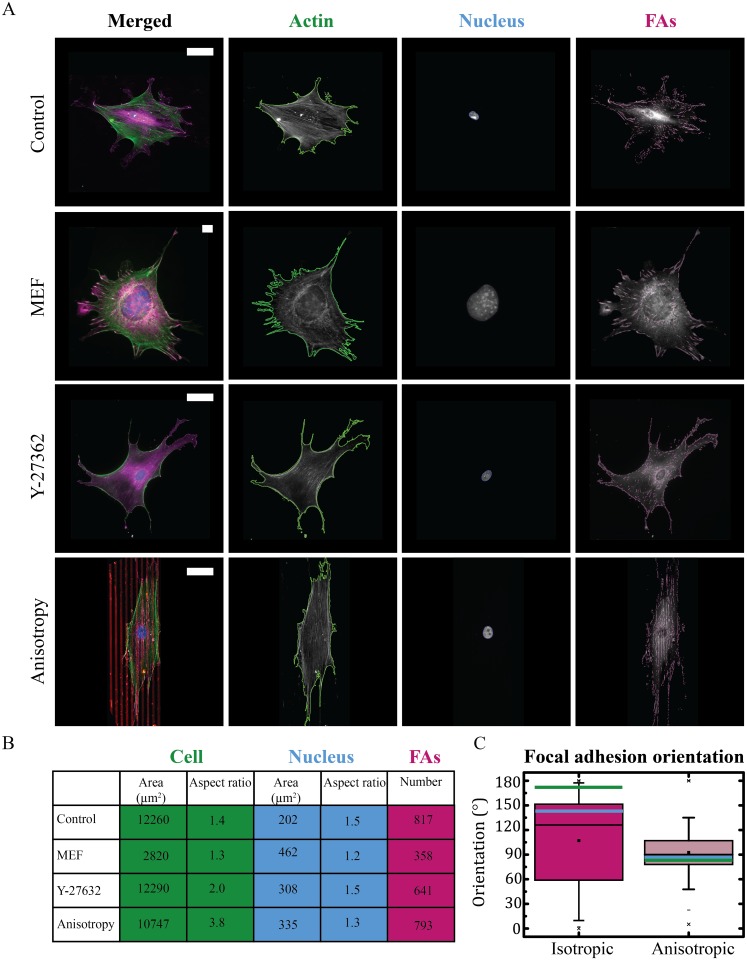
Quantitative analysis of the morphological features of a single cell, nucleus and individual focal adhesions (FAs) obtained under different experimental conditions, e.g. cell type, pharmacological, and substrate manipulation. A: Representative immunofluorescence images of the actin cytoskeleton (green), nucleus (blue), and FAs (magenta) for a HVSC (control) and a MEF (cell type) on a substrate homogeneously coated with fibronectin, HVSC in the presence of 10 μM of Y-27632 (drugs), and HVSC on 5x5 μm lines of fibronectin (red, anisotropy). The detected outlines are shown in green, blue, and magenta respectively. Scale bar: 50 μm. B: Analyzed morphological features of a single cell, and corresponding nucleus and FAs for the control, cell type (MEF), drug (Y-27632) and environment (anisotropy) situation. C: Boxplot of the orientation of the FAs comparing cells cultured on isotropic and anisotropic substrates. The box and whisker plot indicate the median (black line in the box), 25th percentile (bottom line of the box), 75th percentile (top line of the box), and 5th and 95th percentiles (whiskers). Next to this, the orientation of the cell and nucleus are represented by the green and blue lines, respectively. 90° represents the direction of the lines.

Next, we tested the robustness of our algorithm by comparing the morphological features of cells, nuclei and FAs of HVSCs seeded on a substrate homogeneously coated with fibronectin (control) with the extracted features captured under different experimental conditions, e.g. cell type, pharmacological drug, anisotropic environment, as shown in Fig 5A. Anisotropy is a key structural parameter of the physical environment of cells that has an influence on the cellular orientation response [31–33]. We seeded HVSCs on an isotropic (homogeneous) and anisotropic (5x5 μm lines) substrate and we observed that HVSCs align in direction of the anisotropy, orienting their FAs accordingly (Fig 5A). Quantitative analysis of the orientation of the FAs revealed that for a homogeneous substrate more FAs oriented randomly, while for an anisotropic substrate FAs had a preferred orientation along the direction of the anisotropy (Fig 5C). Moreover, the cell morphology was also affected by substrate anisotropy: the aspect ratio was ∼2.5 fold higher for cells on the anisotropic substrate compared to the cells on the homogeneous substrate (Fig 5B), as cells on the homogeneous substrate exhibited a round morphology, while cells on the anisotropic substrate exhibited an elongated morphology. The influence of substrate anisotropy on cellular responses is a well-studied phenomenon with different cell types. To show that our algorithm was able to perform a quantitative analysis on various cell types accurately, we tested our algorithm to detect and analyze a much smaller, widely used cell type, the Mouse Embryonic Fibroblast (MEF) (Fig 5A). The detected outlines acquired from the actin cytoskeleton and FA stained images show that the algorithm was able to detect the small actin protrusions and individual FAs. Quantitative analysis revealed that the morphological features of a cell, nucleus and FAs depends on the cell type (Fig 5B).

To further test the robustness of our algorithm for analyzing FAs, we treated the HVSCs with a Rho-associated kinase (ROCK) inhibitor, Y-27632, which has previously been shown to directly affect FA morphology [34–36]. We observed that when HVSCs were treated for 1 hour with 10 μM Y-27632, the number of central stress fibers was greatly reduced and smaller FAs were observed. Consequently, the actin staining was much weaker and fewer mature FAs were formed. Nevertheless, the algorithm was still able to detect the ROCK-inhibited cell and FAs accurately, as shown by the good agreement of the detected outline acquired from the actin image with the cell boundary (Fig 5A). Taken together, the algorithm was compiled for a variety of experimental conditions, where no need of tweaking of individual parameters was needed. For different factors influencing cellular, nuclear and FA morphological features the algorithm was able to extract morphological changes of individual cells, nuclei and FAs from a relatively heterogeneous data set in a robust way.

### ROCK inhibitor Y-27632 affects FA morphology in a dose-dependent manner

**Fig 6:**
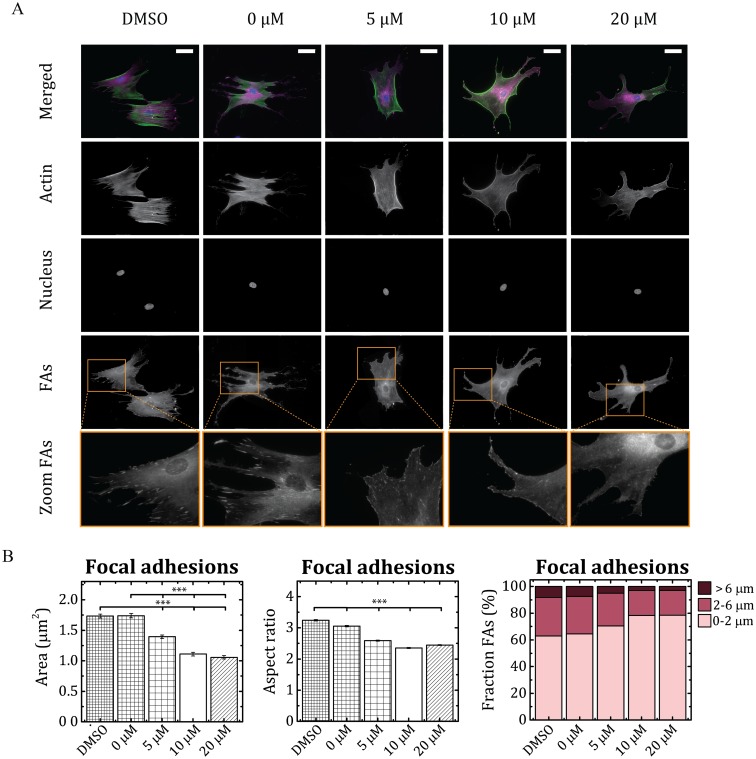
The effect of the Rho-associated kinase (ROCK) inhibitor Y-27632 on the morphological features of focal adhesions (FAs) in Human Vena Saphena Cells (HVSCs). A: Representative immunofluorescence images of the actin cytoskeleton (green), nucleus (blue), FAs (magenta) and zoom-in images of FAs of HVSCs treated with different doses (0-20 μM) of ROCK inhibitor or DMSO (control). Scale bar: 50 μm. Quantitative analysis of FA area (B), FA aspect ratio (C), and fraction of FAs with a defined length (D) reveals that Y-27632 affects FA morphology. At least 20 cells were analyzed per each condition and the results are expressed as the mean ± standard error of the mean (SEM). To assess differences between the different concentrations of ROCK inhibitor on the morphological features of the FAs, the One-Way ANOVA with a Bonferroni post-hoc test was used. ***: p < 0.001.

As a proof-of-principle, we utilized our algorithm to quantify FA morphology at different maturation levels in Human Vena Saphena Cells (HVSCs). When cells adhere to a substrate, they first form small, nascent FAs (0-2 μm-long) which can either disappear or develop into 2-6 μm-long, mature FAs [5]. This transition is driven by mechanical force exerted by contractile actin–myosin stress fibers. Myofibroblasts can develop even larger ‘supermature’ FAs (10-30 μm-long) under conditions of extraordinary high stress, such as stiff substrates [37]. Since FA maturation has been demonstrated to be affected by the ROCK pathway [8, 35], we hypothesize that treatment with varying doses of ROCK inhibitor can lead to different FA maturation states which can be captured quantitatively using our algorithm. To test this, we incubated HVSCs with varying concentrations (0-20 μM) of ROCK inhibitor Y-27632 for 1 hour prior to fixing. For each dose of ROCK inhibitor, 20 cells corresponding to > 7000 FAs per condition were analyzed. As expected, the cell morphology of the HVSCs was clearly affected by the ROCK inhibitor (Fig 6A). Consistent with the characteristic morphology of ROCK-inhibited cells, HVSCs showed more protrusions at high concentrations of Y-27632 (10 and 20 μM) compared to control cells (DMSO). At the same time, they showed less central stress fibers and the associated FAs became smaller and more punctuated. Interestingly, the peripheral stress fibers were hardly affected and the cell size was preserved (S2 Fig). This is consistent with the results reported previously by Katoh et al. [34]. However, with low concentrations of inhibitor (5 μM), central stress fibers were still observed and were found to be connected to larger, elongated FAs [36]. Using our algorithm we were able to quantify the observed effect of Y-27632 on focal adhesion maturation (Fig 6A) by comparing the area of FAs of cells that are treated with various doses of ROCK inhibitor and the FA size in nontreated cells (Fig 6B). Quantitative analysis revealed that treatment with ROCK inhibitor reduced the size of FAs significantly (p <0.001) and that this reduction was dose-dependent. Similarly, quantitative analysis of FA aspect ratio revealed a decrease in aspect ratio for increasing concentrations of ROCK inhibitor (Fig 6C). This shows that with our algorithm we were able to detect changes in the morphological features of FAs in response to ROCK inhibitor, which has an effect on the maturation levels of the FAs. A morphometric determination of different adhesion types can provide helpful information, especially when analyzing dynamic processes, such as cell migration.

As observed in Fig 6A, HVSCs on fibronectin-coated substrates exhibit different sizes of FAs. We classified the FAs in three different groups, nascent (0-2 μm), mature (2-6 μm), and supermature (>6 μm) FAs, based on the FA length. The fraction of FAs in each group for the different doses of ROCK inhibitor was determined (Fig 5D). Consistent with the area of FAs, we found that for a high concentration of ROCK inhibitor, HVSCs exhibited more small, nascent FAs (78%) and less ‘supermature’ FAs (3%) in comparison with the nontreated HVSCs (nascent: 63%, ‘supermature’: 8%). The formation of fully developed, mature FAs requires Rho-kinase activity. Altogether, the quantitative analysis of FAs showed a dose-dependent reduction in maturation of FAs of myofibroblasts in response to ROCK inhibitor Y-27632. This demonstrates the usefulness of our algorithm for providing quantitative insights into the role of FAs in mechanosensing.

### Limitations of the algorithm

With the developed algorithm we were able to successfully detect cellular and nuclear morphology by using a marker-controlled watershed segmentation method to detect single cells and nuclei. This approach has a limitation, detection of individual cells in a confluent monolayer can not be performed effectively. We found more object markers than actual cells, resulting in over-segmentation and false positive objects. The main reason of this is that the intensity variations in the actin staining along the touching/overlapping cells was too small to be detectable and thus to separate cell clusters. To address this limitation, images where the plasma membrane is stained, for example with CellMask (Thermo Fisher Scientific, Waltham, USA), could be beneficial for watershed-based segmentation of cells [38]. In addition, detection of FAs of multiple, touching cells within one image can also be a limitation, as the algorithm could not be fully automated but needed manual tracing of the cell. This way of analyzing is more time-consuming, but will lead to more robust detection of individual FAs in the case of touching cells.

## Conclusion

Our work presents a straightforward segmentation strategy to automatically and accurately process raw images of the actin cytoskeleton, nucleus, and focal adhesions to detect individual (sub)cellular components. The automated algorithm is particularly useful for obtaining high-throughput quantitative (sub)cellular data relevant to identify important morphological changes between different cell types and in response to different environmental or pharmacological manipulations. A full, open-source software implementation of this pipeline is provided to contribute to further research on the mechanisms of how cells sense and respond to different environmental properties.

## Supporting information

S1 Table Comparison between different published methods. (PDF)

S1 Fig Focal adhesion length for 0 μM and 10 μM of Rho-associated kinase inhibitor Y-27632. A) The fraction of focal adhesions (FAs) of a specific length determined for all analyzed cells. To make sure that the algorithm only detects actual FAs, a threshold for the length of individual FAs was determined. The dashed line represents the threshold of 30 μm. B) Representative immunofluorescent image of FAs overlaid with the detected outlines in magenta and zoom-in image, where the arrow indicates a false detection.
(TIF)

S2 Fig Morphological features of cells and nuclei for HVSCs treated with 10 μM of Rho-associated kinase inhibitor Y-27632 and nontreated HVSCs (DMSO). Quantitative analysis of cells and nuclei reveals that Y-27632 affects the cellular and nuclear aspect ratio and area of the nuclei. The results are expressed as the mean ± standard error of the mean (SEM). The differences for cell and nucleus area and aspect ratio between the DMSO and 10 μM of ROCK inhibitor situation was assessed by using an independent sample t test (normal) or Mann- Whitney U test (non normal). *: p < 0.05, ***: p < 0.001.
(TIF)

S1 File Custom-made code written in Mathematica 11.1 for the analysis of cell, nucleus and focal adhesion morphology. (NB)
