# Supplementary material for: An automated quantitative analysis of cell, nucleus and focal adhesion morphology
Source: PLoS One. 2018 Mar 30;13(3):e0195201. doi: 10.1371/journal.pone.0195201 (PMC5877879; doi:10.1371/journal.pone.0195201)
Supplement: S1 Table — (PDF) [file pone.0195201.s001.pdf]

	Berginski et al.	Broussard et al.	Carpenter et al.	Horzum et al.	Würflinger et al.	Buskermolen et al.
**Properties**						
Segmentation	v	v	v	x	v	v
Automated	v	v	x	x	v	v
Batch processing	v	v	v	x	v	v
Open source	?	?	v	v	x	v
Robustness	v	v	x	x	v	v
**Detectable features**						
Cell	x	x	v	x	x	v
Nucleus	x	x	v	x	x	v
FAs	v	v	v	v	v	v

FAs = focal adhesions
