# Supplementary figures and images for: An automated quantitative analysis of cell, nucleus and focal adhesion morphology
Source: PLoS One. 2018 Mar 30;13(3):e0195201. doi: 10.1371/journal.pone.0195201 (PMC5877879; doi:10.1371/journal.pone.0195201)

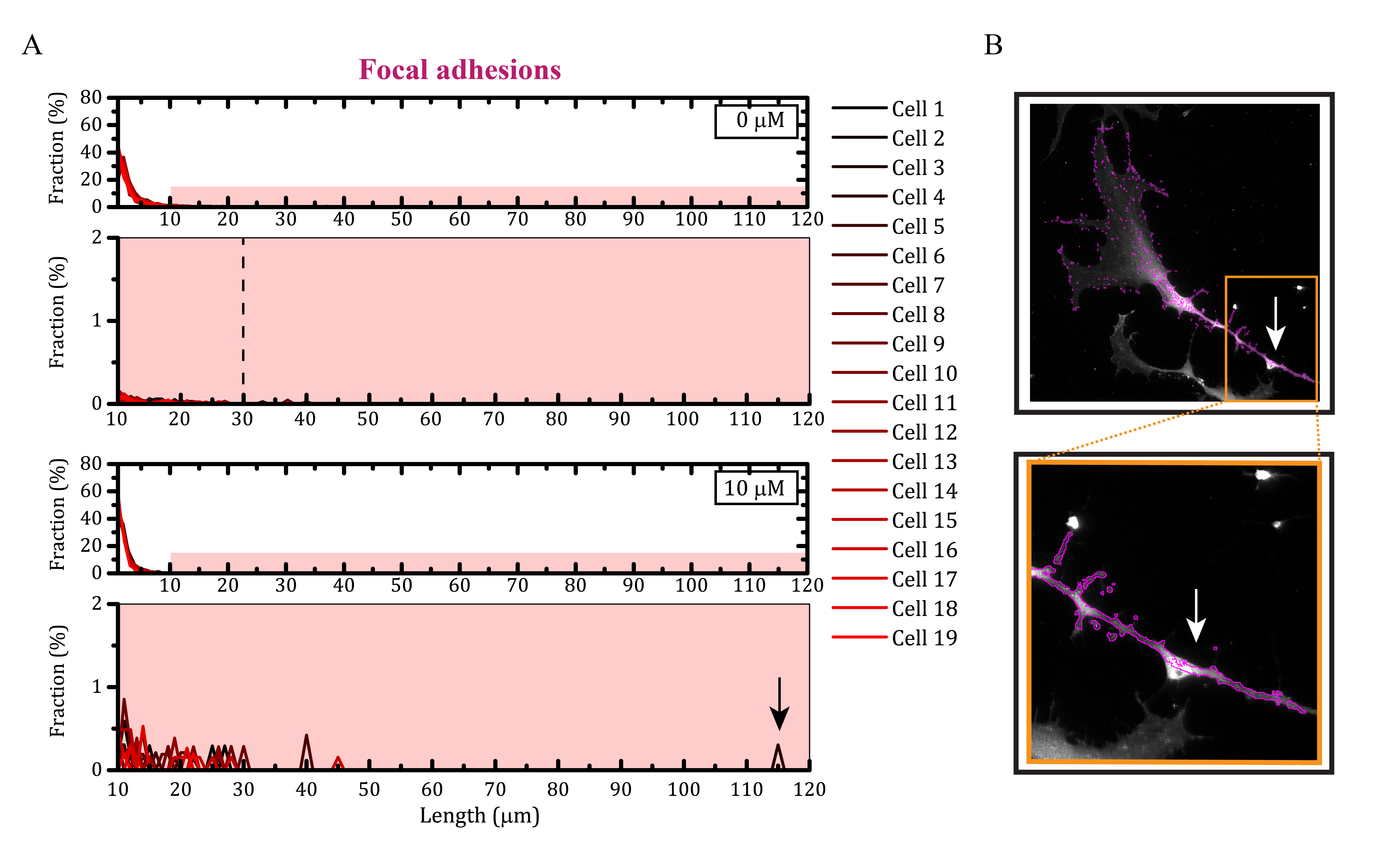

Supplement: S1 Fig — A) The fraction of focal adhesions (FAs) of a specific length determined for all analyzed cells. To make sure that the algorithm only detects actual FAs, a threshold for the length of individual FAs was determined. The dashed line represents the threshold of 30 μm. B) Representative immunofluorescent image of FAs overlaid with the detected outlines in magenta and zoom-in image, where the arrow indicates a false detection. (TIF) [file pone.0195201.s002.tif]

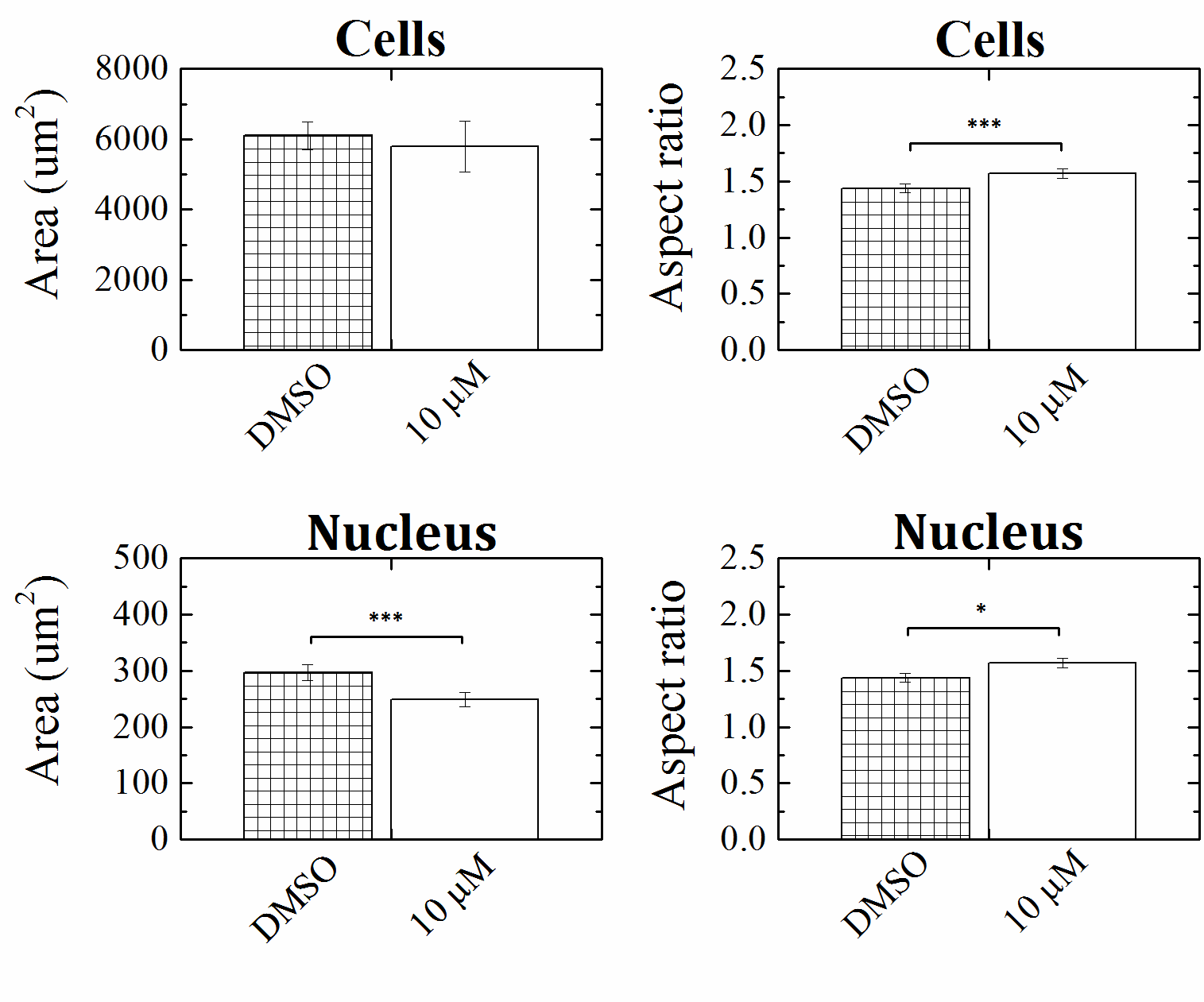

Supplement: S2 Fig — Quantitative analysis of cells and nuclei reveals that Y-27632 affects the cellular and nuclear aspect ratio and area of the nuclei. The results are expressed as the mean ± standard error of the mean (SEM). The differences for cell and nucleus area and aspect ratio between the DMSO and 10 μM of ROCK inhibitor situation was assessed by using an independent sample t test (normal) or Mann- Whitney U test (non normal). *: p < 0.05, ***: p < 0.001. (TIF) [file pone.0195201.s003.tif]
